# Real-Time Fibrinolysis Monitoring of Plasma Annular Clots

**DOI:** 10.3390/biom16070949

**Published:** 2026-06-26

**Authors:** Andres Prieto Trujillo, Anushri Umesh, Abigail Hall, Nathan J. Alves

**Affiliations:** 1 Department of Emergency Medicine, Indiana University School of Medicine, 635 Barnhill Dr. Rm. 2063, Indianapolis, IN 46202, USA; 2 Weldon School of Biomedical Engineering, Purdue University, West Lafayette, IN 47907, USA

**Keywords:** fibrinolysis assessment, plasma clots, fluorescence, thrombolytic therapy

## Abstract

Fibrinolysis assessment is critical for diagnosing and managing clinical blood disorders. Currently available viscoelastic testing platforms provide an overview of global coagulation and fibrinolysis profiles but lack fibrinolysis specific assessment of a preformed clot substrate. The lack of a sensitive, standardized testing platform for fibrinolysis assessment can limit risk stratification and the management of blood disorders. We describe herein the plasma annular clot lysis assay. The assay adapts the previously standardized FITC-tagged fibrin annular clot assay to plasma obtained from healthy human donors. Plasmin at concentrations ranging from 200–800 nM was used as a direct thrombolytic to assess fibrinolysis in plasma-derived annular clots. The Maximum Fibrinolysis Rate (V_MFR_), FLU200, T90, and MaxFLU were calculated via tracking of the clot digestion curve over time. V_MFR_ was correlated with plasma thromboelastography (TEG) parameters to compare with the global viscoelastic testing system. Additionally, plasma annular clot digestion was monitored in the presence of pentamidine to assess drug-specific effects on fibrinolysis tracking. Plasma annular clots linearly tracked fibrinolysis with increasing plasmin concentration. V_MFR_ across multiple tested plasmin concentrations showed a moderate to strong negative correlation (0.58–0.74) with the observed maximum amplitude from plasma TEG. In the presence of pentamidine (75 µM), the assay was sensitive to identifying differences in the V_MFR_ across treatment groups. Plasma annular clots provide a platform for fibrinolysis evaluation using a patient’s own plasma to assess therapeutic dosing clinically in addition to testing novel therapeutics preclinically to further understand mechanistic aspects of fibrinolysis.

## 1. Introduction

A comprehensive understanding of the interplay between coagulation and fibrinolysis is essential for diagnosing and managing a wide spectrum of clinical disorders, including sepsis, disseminated intravascular coagulation (DIC), trauma-induced coagulopathy, hereditary bleeding disorders, and thrombotic diseases [1,2,3]. Coagulation testing platforms have evolved rapidly, with viscoelastic platforms such as Thromboelastography (TEG), Rotational Thromboelastography (ROTEM), and ClotPro providing a global, real-time assessment of clot formation and fibrinolysis in whole blood [4,5,6]. However, traditional fibrinolysis assessment relies on endpoint tests, such as blood D-dimer levels or LY30 from global assays, to characterize fibrinolysis. These methods are known to not capture subtle fibrinolytic alterations, leading to high variability hampering risk stratification in conditions such as sepsis [7,8]. Though newer methods, such as clot-fibrinolysis waveform analysis (CFWA), show promise as a more sensitive assay to capture fibrinolysis, especially in DIC, they remain to be clinically validated for their applicability [9,10,11]. The fundamental challenge in this field is that fibrinolysis in vivo is a slow, tightly regulated process, and fibrinolytic activators must typically be added or inhibitors reduced to induce measurable fibrinolysis within a clinically relevant time frame [4,12]. Therefore, validation and standardization of methods to measure fibrinolysis in vitro have progressed more slowly than for coagulation testing, and widely accepted assays remain unavailable [4]. Studies have strongly emphasized that available methods fail to robustly distinguish hyperfibrinolysis, physiological fibrinolysis, hypofibrinolysis, and fibrinolysis shutdown in clinical settings [7,8]. Since fibrinolysis phenotypes have differential therapeutic implications (e.g., TXA vs. fibrinolytics), the lack of reliable, phenotype-specific readouts limits targeted therapy decisions [7,8,13]. Fibrinolysis assessment in vitro and ex vivo remains challenging, with studies employing euglobulin clot lysis time, plasma clot lysis time, and fluorogenic plasmin generation yielding inconclusive or conflicting results [12]. This substantial variability across assays, platforms, and laboratories underscores the need for assays that standardize readouts to improve comparability, which led to the development of our previously reported FITC-tagged fibrinogen annular clot fibrinolysis assay [14].

The fibrinolytic system is tightly regulated and is initiated when plasminogen is converted to active plasmin by tissue plasminogen activator (t-PA) or urokinase plasminogen activator (u-PA). The balance between coagulation and fibrinolysis is maintained by serine protease inhibitors (SERPINs) such as Plasminogen Activator Inhibitor 1 and 2 (PAI-1, PAI-2) and alpha-2-antiplasmin (α2-AP), which inhibit t-PA, u-PA, and plasmin, respectively [15,16]. Given the complexity of this regulatory system, differences in levels of endogenous fibrinolysis inhibitors are linked to major adverse cardiovascular events and bleeding risks [17,18,19]. The shifts in these fibrinolysis inhibitor levels are known to cause downstream vascular dysfunction and increased thrombotic risk by impairing fibrinolysis and altering clot architecture [20]. To better characterize the complex interplay among proteases and inhibitors, a sensitive, reproducible assay system is necessary. While the previously developed annular clot lysis assay provided a standardized fibrin clot substrate to compare digestion rates across samples, this work extends the assay to utilize plasma derived from healthy donors to test clinical fibrinolytic-based interventions.

**Figure 1:**
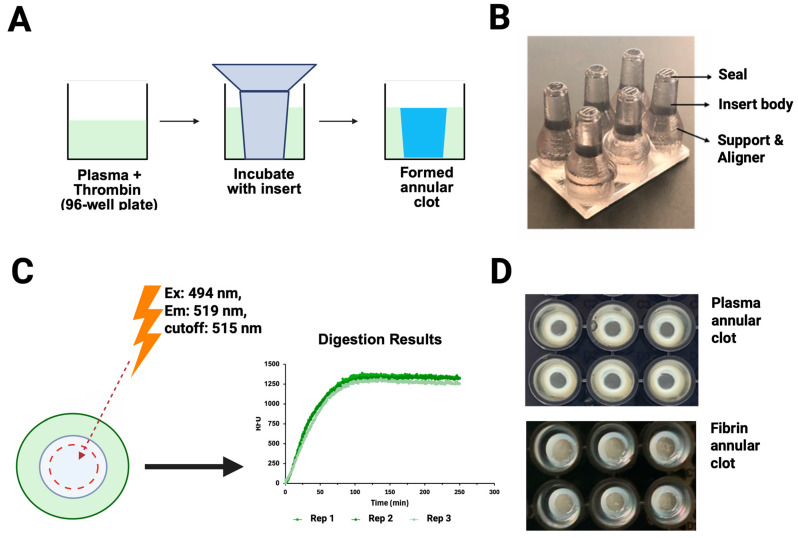
(**A**) Schematic of formation steps needed to create the FITC-tagged plasma annular clot. (**B**) Image of the annular clot insert. (**C**) Parameters used for mapping digestion and the representative digestion curve. (**D**) Picture of formed plasma and fibrin annular clots multiplexed in a 96-well plate. (Figure created using BioRender).

Enabling monitoring of multiple patient plasma samples, or multiple different assay conditions with the same patient’s plasma, provides an advantage of screening multiple thrombolytic conditions simultaneously to improve testing throughput. The annular clot assay described previously [14] enables real-time measurement of fibrinolysis using a simplified fibrin annular clot. The fibrin annular clot lysis assay was previously tested to reliably measure kinetic parameters in pre-formed, labeled fibrin clots. At a 1:50 FITC-Fibrinogen-to-native fibrinogen concentration, we observed minimal impact of the reporter FITC-labeling on the overall microarchitecture of the clot, and the fibrin annular clot enabled dynamic, real-time tracking of clot digestion [14]. At varying concentrations of plasmin and t-PA, the fibrin clot produced sensitive and reproducible kinetic measurements, including Fluorescence Release Rate (V_FR_) and FLU200. The FITC-tagged fibrin annular clot provided a standard substrate for measuring fibrinolysis in the presence of exogenous activators or inhibitors; however, it is a simplified system compared to plasma. Plasma contains many native proteins that assist in clot formation, such as Factor XIII, which can increase clot strength through crosslinking and impact fibrinolysis [21]. Additionally, as previously described, plasma also contains varying concentrations of fibrinolysis inhibitors, such as PAI-1, PAI-2, α2-AP, and TAFI, which directly and indirectly regulate fibrinolysis [15,16]. This study explores the adaptation of the standardized fibrin annular clot lysis assay to plasma annular clots formed from healthy human donors. The formation of a plasma annular clot and a representative image of the fibrin and plasma annular clot are shown in Figure 1. Kinetic parameters obtained from the plasma annular clot are compared to the previously reported fibrin-based annular clot assay to identify differences between the two systems. The Maximum Fibrinolysis Rate (V_MFR_), a key parameter indicating lytic potential, is also compared with the Maximum Amplitude (MA) from plasma thromboelastography (TEG) to assess the correlation between observed global TEG parameters and kinetic parameters obtained from the plasma annular clot lysis assay. Plasma MA reflects overall clot strength, which is driven by fibrin cross-linking [22,23,24,25]. Characterizing the relationship between these two parameters may therefore provide insights into how clot strength relates to its susceptibility to fibrinolytic breakdown. Additionally, the lysis potential of the plasma annular clot was studied in the presence of pentamidine. Pentamidine has previously been shown to inhibit multiple serine proteases central to hemostasis and fibrinolysis, including plasmin, thrombin, and t-PA, with inhibition constants in the micromolar range [26,27,28,29]. Therefore, Pentamidine was used as a representative inhibitor in the annular clot assay to screen for effects on fibrinolysis kinetics in the presence of an exogenous serine protease inhibitor.

## 2. Materials and Methods

### 2.1. Blood Collection and Isolation of Platelet-Poor Plasma

Healthy human blood for research was obtained from consented adult donors (n = 10: 5 males; 5 females) under an approved IRB (protocol #1610652271) at Indiana University School of Medicine. 30 mL of blood was drawn intravenously into citrated BD Vacutainer tubes (BD #363083). A 400 µL aliquot of blood was used for whole blood thromboelastography. Citrated blood was transferred to 15 mL polypropylene tubes and centrifuged at 25 °C for 15 min at 2000× *g* to generate platelet-poor plasma (PPP). All donor blood and plasma were processed and stored at room temperature, and all analyses were performed on the same day as collection.

### 2.2. Thromboelastography of Blood and Plasma Samples

To assess global coagulation and fibrinolysis profiles, thromboelastography (Haemonetics TEG^®^ 5000, Haemonetics Corporation, Boston, MA, USA, #07-022) was performed in non-kaolin-activated TEG cups, with the addition of 340 µL blood or plasma, respectively, and 20 µL 200 mM CaCl_2_ to initiate clotting. TEG runs were measured for 60 min to obtain TEG values for both blood and plasma samples.

### 2.3. Preparation of Fluorescently Tagged Fibrinogen

Fluorescein isothiocyanate (FITC; Sigma Aldrich, St. Louis, MO, USA, #46950) and human fibrinogen (Sigma Aldrich, St. Louis, MO, USA, #341578) were used to generate FITC-tagged fibrinogen using the method previously described in Zeng et al. [14] In brief, human fibrinogen was hydrated in PBS, incubated with FITC at a 1:200 fibrinogen-to-FITC ratio, protected from light, and reacted at room temperature overnight. Conjugated FITC Fibrinogen was purified by centrifugation through 100 kDa filters and washed until all excess FITC was removed. Total conjugation level was calculated by measuring absorbance at 280 nm and 494 nm correcting for the FITC absorbance contribution at 280 nm, using the Spectromax M5E plate reader ((Molecular Devices, San Jose, CA, USA), and the average conjugation level was calculated to be 12-FITC conjugations per fibrinogen. Fluorescence was quantified at Ex: 494 nm, Em: 519 nm, cutoff: 515 nm.

### 2.4. Fibrin and Plasma Annular Clot Formation

The methods for fabricating purified fibrin annular clots are detailed in a previous study [14]. Briefly, 75 µL of fluorescently labeled and unlabeled fibrinogen clotting solution (total of 3.2 mg/mL) was added to microplate wells, and clotting was initiated with 5 µL of 16 U/mL of human thrombin (Haematologic Technologies Inc., Essex Junction, VT, USA, #HCT0020); immediately, the 3D printed well insert (Figure 1B) was pressed into the well and incubated at room temperature for 30 min. As for plasma annular clots, the 96-well microplates (Corning #3601, Sigma Aldrich, St. Louis, MO, USA, #CLS3601) were preheated to 37 °C on a metal heat block. FITC reporter-labeled clots were formed by spiking a 50:1 ratio of naive human fibrinogen to 12FhF to produce a fluorescent fibrinogen clotting solution. For plasma clots, the average native fibrinogen concentration was estimated to be 3.2 mg/mL. The plasma sample was brought to a final concentration of 11 mM CaCl_2_ to initiate clotting, and the microplate inserts were immediately pressed into the well and incubated at 37 °C for 1.5 h to ensure formation of a stable plasma clot. After clotting was complete, both plasma and fibrinogen annular clots were washed several times with PBS to remove any unreacted material. All clots were submerged in 120 µL of PBS and inspected via an endpoint fluorescence read (SpectraMax M5E); Ex: 494 nm, Em: 519 nm; 1000 RFU cutoff) as well as visually for any detachment from the well or occlusion of the center path malformations. Annular clots remained submerged in PBS, away from light, at room temperature prior to use.

### 2.5. Fibrinolysis Assay

Lyophilized human plasmin from plasma (Athens Research & Technology, Athens, GA, USA, #16-16-161213-L) was reconstituted in PBS and diluted to experimental concentrations of 200, 350, 500, 650, and 800 nM (0.17–0.67 U/mL); plasmin activity was standardized using the soluble substrate S-2251 (DiaPharma Chromogenix S-2251, West Chester, OH, USA, #S820332) activity assay. The activity was within the range of the maximum inducible plasmin activity of 1 U/mL in blood [30]. Fibrinolysis assays were initiated by adding 120 µL plasmin solution to the center of an annular clot well and monitoring using a SpectraMax M5E plate reader (Ex: 494, Em: 519) for 4.5 h, or until no increase in fluorescent signal was observed. Fibrinolysis by plasmin at different concentrations was measured in both fibrin and plasma annular clots, with 4 replicates per condition (n = 10 donors). In a separate experiment, plasma annular clots were tested at the same plasmin concentrations but pre-incubated with 75 μM Pentamidine to screen for drug-specific activity. Fibrinolysis dynamics were analyzed and compared based on the maximum fibrinolysis rate (V_MFR_). The V_MFR_ was determined using a dynamic sliding-window linear regression applied to raw fluorescence-time kinetic curves. Linear models were fitted over overlapping 10–20 min windows, and the window with the highest R-squared was taken as the V_MFR_. Additional parameters, including time to reach 90% (T90), maximum fluorescence (MaxFLU), and time to a minimum detection threshold of 200 RFU (FLU200), were compared to screen inter-group differences.

### 2.6. Statistical Analysis

A log-transformed linear mixed model (Log-LMM) was used to analyze the effect of increasing plasmin concentration on V_MFR,_ with each donor (n = 10: 5 males; 5 females) treated as a random effect across all datasets to account for repeated measures at different concentrations. Given the limited sample size (n = 5 per sex), sex was included as a covariate in the linear mixed model to adjust for confounding rather than to draw inferential conclusions regarding sex-specific differences in fibrinolytic kinetics. Type III ANOVA assessed fixed effects, and significant differences between the groups were evaluated using pairwise post hoc comparisons, with marginal means estimated and Tukey adjustments applied. The Wilcoxon rank-sum test with Benjamini–Hochberg (BH) correction was used as a nonparametric test to compare FLU200, MaxFLU, and T90 across fibrin and plasma clot datasets. Additionally, Wilcoxon signed-rank tests were used to compare the plasma and pentamidine-treated groups. Spearman’s rank correlation was used to assess the association between maximum digestion rate and MA value from TEG at each plasmin concentration, with Benjamini–Hochberg correction for multiple comparisons. Adjusted *p*-values < 0.05, 0.01, and 0.001 were considered significant across the study.

## 3. Results

### 3.1. Subject Demographics and Plasma Characterization Using TEG

**Figure 2:**
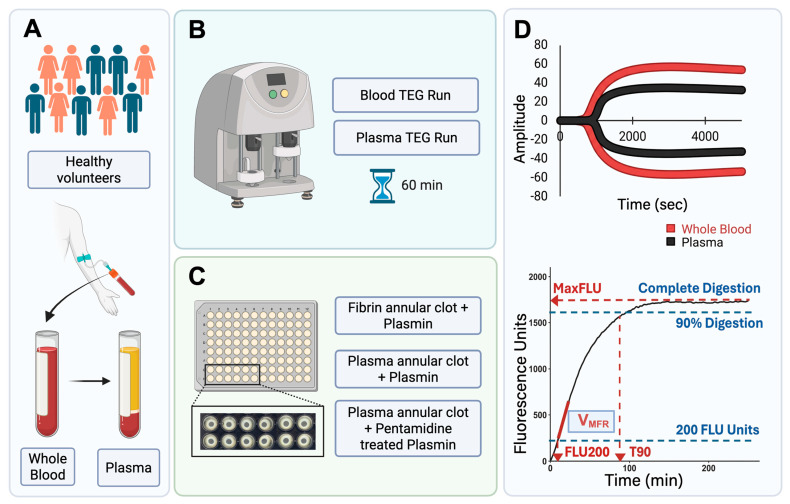
Overall study design for plasma annular clot design and analysis. (**A**) Collection of blood from healthy donors and subsequent platelet-poor plasma generation. (**B**) Thromboelastography run for blood and plasma to characterize the global coagulation and fibrinolysis profile. (**C**) Fibrin and Plasma spiked with FITC-Fb were digested under varying concentrations, only plasmin and plasmin in the presence of pentamidine. (**D**) Representative graphs for thromboelastography and annular clot fibrinolysis profile carried out in the study. (Figure created using BioRender).

A total of 10 healthy donors (5M/5F) were enrolled in the study. The mean age of the cohort was 25.8 ± 5.9 years. An overview of the study design is shown in Figure 2. 30 mL of whole blood was collected from each subject, and plasma was isolated via centrifugation. Plasma was subsequently aliquoted for TEG and downstream annular clot lysis analysis. Both recalcified blood and plasma formed clots for all tested donors within 60 min of the TEG analysis run. Baseline TEG values for non-kaolin-treated whole blood and plasma were analyzed, and are reported in Tables S1 and S2, respectively, and TEG curves are presented in Figure S1. The plasma TEG values had a lower MA than the whole-blood TEG values, as expected, because the platelet contribution to the maximum amplitude is absent in the plasma TEG samples. This plasma, with the addition of FITC-tagged fibrinogen (FITC-Fb), was subsequently used to generate all downstream annular clots, which were digested at varying plasmin concentrations to evaluate lysis kinetics. The volume of plasmin solution required for the assay to calculate V_MFR_ has been optimized, and the velocities at different plasmin solution volumes in the annular clot lysis assay are shown in Figure S2.

### 3.2. Kinetic Comparisons of Fibrin and Plasma Annular Clots

The initial analysis of the study focused on the fundamental characterization of plasmin-mediated fibrinolysis in fibrin and plasma annular clots. Plasma annular clots were compared with fibrin annular clots formed using the methodology previously described in Zeng et al. [14] Plasma clots were formed, spiked with an equivalent ratio of FITC-Fb as fibrin clots, and incubated at 37 °C for a longer period to fully stabilize the clots. Plasmin was introduced across a concentration range of 200 to 800 nM to both fibrin and plasma annular clots to establish fibrinolytic curves with FITC-Fb as the reporter. A sliding-window linear regression was applied to the kinetic curves to identify the maximum slope (RFU/min), which was considered to be the Maximum Fibrinolysis Rate (V_MFR_) in this study. Additional parameters screened in the study include FLU200, defined as the time required to reach release of 200 fluorescence units in the digestion assay, and MaxFLU, the maximum fluorescence units reached in each well by the end of clot digestion. T90 is calculated as the time at which the fluorescence release reaches 90% of the MaxFLU observed in individual digested clots, corresponding to the time needed to digest the clot by 90%. Values for all parameters obtained from annular clot lysis analysis are provided in Table S3.

Screening for the above parameters, fibrin annular clots were digested with increasing plasmin concentrations ranging from 200–800 nM across seven replicates to establish the reproducibility of fibrinolysis rates in the assay. This was also used as the baseline to confirm the ability of the assay to capture dose-dependent fibrinolysis with increasing plasmin concentrations. The primary model used to study the main effects of plasmin concentration on V_MFR_ was a log-transformed linear mixed model (Log-LLM). Normality assessment before and after log transformation for all analyzed dataset is shown in Figure S3. The main effects from Log-LLM revealed that the V_MFR_ was significantly affected by increasing plasmin concentration (F = 988.8, *p*-value < 0.001), with the average maximum fibrinolysis rate increasing from 2.8 ± 0.6 RFU/min at 0 nM to 55.9 ± 12.2 RFU/min at 800 nM. Pairwise comparisons (Tukey-adjusted) confirmed a significant difference between baseline (0 nM) and all plasmin concentrations, with adjusted *p*-values < 0.001.

Consequently, V_MFR_ was measured in plasma samples from 10 healthy donors at identical plasmin concentrations, as determined in a fibrin annular clot. This dataset demonstrates the adaptation of annular clots for use with human plasma samples. Minimal baseline fibrinolysis was observed across both fibrin and plasma annular clots when incubated with PBS (Figure S4). Similar to the fibrin annular clot analysis, log-LLM was used as the primary model to analyze the effect of increasing plasmin concentration on V_MFR_. The resulting dose–response profiles demonstrated a statistically significant increase in fibrinolysis with increasing plasmin concentration (F = 166.7, *p*-value < 0.001). Inferences of the main effect of sex were limited due to the smaller sample size in the current analysis. However, on average, a qualitative trend was observed with plasma annular clots from male subjects having a higher fibrinolysis rate compared to female subjects. This difference was evident as the plasmin concentration increased beyond 500 nM. Pairwise comparisons of fibrinolysis rates of plasma clots across different plasmin concentrations showed a significant difference in V_MFR_ starting at 350 nM of plasmin concentration compared to baseline.

**Figure 3:**
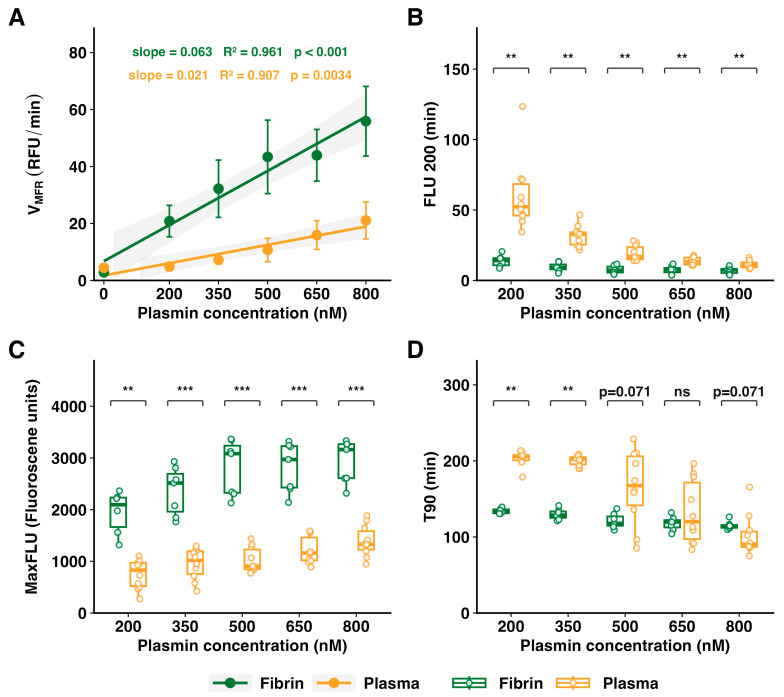
Comparison of fibrin and plasma annular clots. (**A**) Maximum Fibrinolysis Rate (V_MFR_) observed after the 200 RFU threshold. (**B**) FLU200, defined as the time required to reach 200 fluorescence units. (**C**) Maximum fluorescence release measured across a range of plasmin concentrations (200–800 nM). (**D**) Time required to achieve 90% of MaxFLU release in fibrin versus plasma clots. Significance levels of adjusted *p*-value: ** *p* < 0.01; *** *p* < 0.001; ns, not significant; respectively.

Comparing the fibrinolysis of plasma and fibrin annular clots, the fibrinolysis rates remained linear with increasing plasmin concentration across both assays. Plasma and fibrin clots; however, differed in the slope of fibrinolysis, with a 3-fold reduction in the fibrinolysis rate of plasma clots (Slope = 0.02, R^2^ = 0.91) when compared to fibrin clots (Slope = 0.06, R^2^ = 0.96). There was a significant difference in the time required to reach 200 fluorescence units (FLU200) between the fibrin and plasma annular clots at all tested plasmin concentrations, with plasma annular clots requiring significantly longer time to reach FLU200. The difference in values for FLU200 between plasma and fibrin annular clots decreased with increasing plasmin concentration (Figure 3B). Additionally, a significant difference in the MaxFLU was observed between the fibrin and plasma annular clots. The MaxFLU for the fibrin annular clot had a median of 3161.8 [IQR 2607.8–3267.1] RFU, while plasma annular clots had a lower MaxFLU overall, with the highest fluorescence readings reaching up to 1330.5 [IQR 1226.8–1584.4] RFU at an 800 nM plasmin concentration. At lower plasmin concentrations, there were significant differences in fibrinolysis rate: plasma clots treated with 200 nM and 350 nM plasmin required much longer to reach 90% digestion. However, increasing the plasmin concentration tended to narrow this difference between plasma and fibrin annular clots. While the mean velocity scaled linearly with plasmin concentration (Figure 3A), the absolute variance tended to increase at higher plasmin concentrations in plasma annular clots. Despite the expansion in variance, the signal-to-noise ratio remained high enough to distinguish fibrinolysis rates between different plasmin concentrations for both fibrin and plasma annular clots, as confirmed by pairwise comparison of maximum fibrinolysis rate at different plasmin concentrations. This ensured that the maximum fibrinolysis rate we used for subsequent TEG correlation was a stable, reproducible metric of the lytic potential of the plasma annular clots.

### 3.3. Sex-Specific Trends in Maximum Fibrinolysis Rate

**Figure 4:**
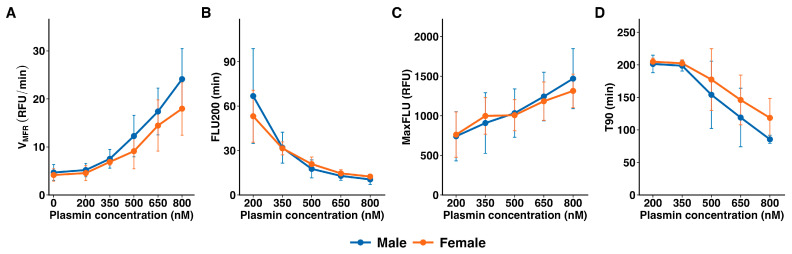
Plasma annular clot lysis kinetics in healthy male and female donors. (**A**) Maximum Fibrinolysis Rate (V_MFR_) observed after the 200 RFU threshold; (**B**) FLU200; (**C**) MaxFLU; (**D**) T90 in male versus female samples.

A sex-stratified kinetic analysis was conducted to identify qualitative trends in the influence of sex on fibrinolysis rates in plasma annular clots. No statistical inferences were drawn due to the limited sample size of 5 males and 5 females in this study. The V_MFR_ in both sexes showed identical dose-dependent kinetic trajectories as measured by the plasma annular clot digestion assay (Figure 4A). The male cohort had a slope of 0.032 (R^2^ = 0.91), and the female cohort had a slope of 0.022 (R^2^ = 0.91). A general trend was observed that the male cohort had a relatively higher fibrinolysis rate than the female cohort, particularly at higher plasmin concentrations. There was no difference in FLU200 and MaxFLU between the male and female cohorts in this limited dataset (Figure 4B,C). Additionally, the male cohort needed less time to reach T90 compared to the female cohort. However, additional samples have to be analyzed to screen if this trend maintains across larger datasets (Figure 4D).

### 3.4. Correlation Between Maximum Fibrinolysis Velocity and Thromboelastography

**Figure 5:**
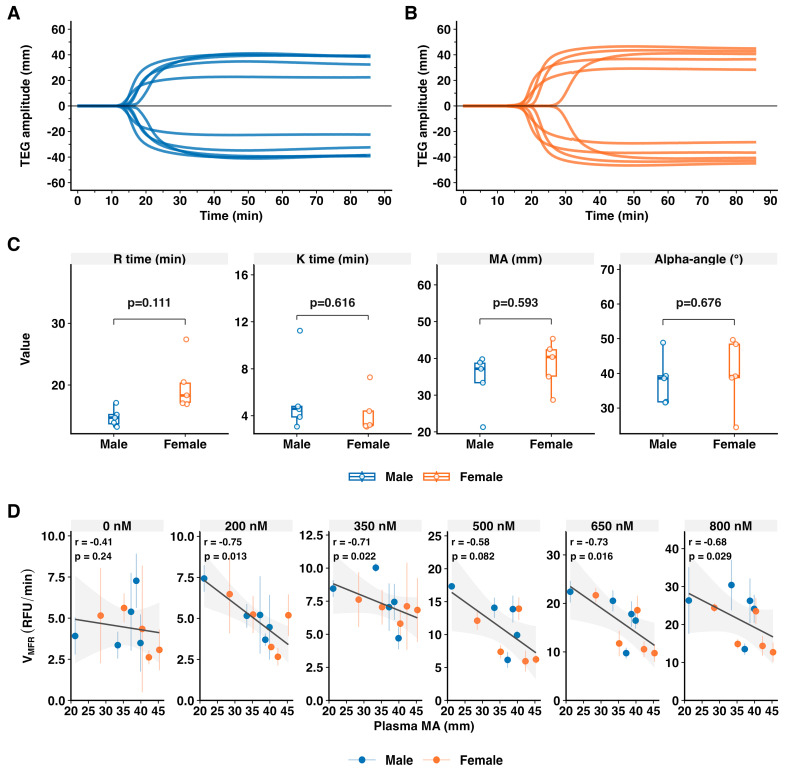
Correlation between TEG and Plasma annular clot analysis. Plasma TEG curves for (**A**) male and (**B**) female cohorts. TEG values (**C**) R time, K-time, Maximum amplitude, and Alpha-angle sex-stratified for male and female cohorts. (**D**) Correlation of TEG parameter maximum amplitude with the maximum fibrinolysis rate of plasma annular clots.

Thromboelastography was performed on all 10 donors to characterize baseline viscoelastic properties of whole blood and plasma in non-kaolin-activated TEG cups. The overall TEG curves for platelet-poor plasma samples for males and females are shown in Figure 5A and Figure 5B, respectively. The summary of calculated TEG parameters is provided in Table S2. Screening the TEG parameters across plasma samples revealed no statistically significant differences between male and female participants under the Wilcoxon test, with BH adjustment for any measured TEG parameters (Figure 5C). However, qualitative screening showed that the overall R time and maximum amplitude were higher in female subjects. Since platelets have been removed from the samples, this observation can be considered specific to clot strength, with the primary contribution coming from overall fibrin strength. Additionally, the MA obtained from TEG analysis was correlated with the V_MFR_ of plasma annular clots. Since V_MFR_ is the primary indicator of lytic potential across different plasmin concentrations, Spearman’s correlation analysis was used to assess the correlation between annular clot lytic potential and overall clot strength. Correlation analysis revealed a moderate to strong inverse relationship between the maximum fibrinolysis rate and clot strength, as measured by TEG Maximum amplitude (MA), at each plasmin concentration, with all correlations having *p*-values < 0.05, except for 500 nM, which had *p*-values of 0.082 (Figure 5D). Across all tested donors, higher plasma MA values were consistently associated with lower maximum fibrinolysis rates. This negative correlation was maintained across plasmin concentrations of 200–800 nM, indicating a dose-independent relationship between plasma clot strength and the maximum fibrinolysis rate in the kinetic assay. 

### 3.5. Application of Plasma Annular Clot Assay to Study Drug-Specific Response

**Figure 6:**
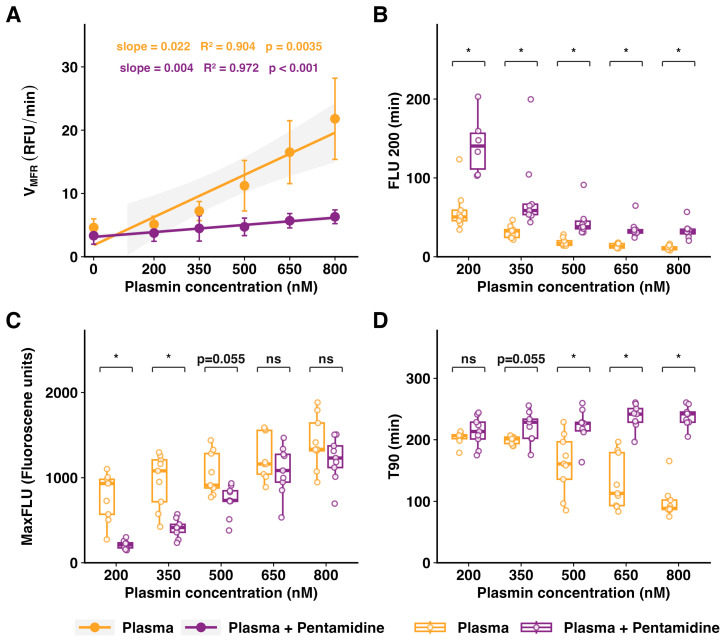
Analysis of plasma annular clot lysis kinetics by plasmin in the presence and absence of pentamidine (**A**) Maximum Fibrinolysis Rate (V_MFR_) for plasma and pentamidine-treated plasma clots. (**B**) FLU200, defined as the time required to reach 200 fluorescence units. (**C**) Maximum fluorescence release measured across a range of plasmin concentrations (200–800 nM). (**D**) Time required to achieve 90% clot digestion. * denotes significance at adjusted *p*-value < 0.05; ns, not significant; respectively.

To evaluate if plasma annular clots can effectively assess drug-specific responses, we compared clot lysis in the presence and absence of pentamidine, a known reversible serine protease inhibitor [28]. Given that one-third of the blood plasma enzymes are serine proteases and that plasmin is also a serine protease, this test aimed to explore the ability of the plasma annular clot assay to capture differences in fibrinolysis responses in the presence of a known serine protease inhibitor. The plasma annular clots remained reproducible even in the presence of pentamidine at 75 µM, with fibrinolysis rates increasing linearly with increasing plasmin concentrations (Figure 6A). The FLU200 time significantly differed between the plasmin and the pentamidine-treated groups at all tested plasmin concentrations (adjusted *p*-value < 0.01) (Figure 6B). At 200 nM plasmin concentration, the FLU200 was between 52.3 [IQR 46.0–68.4] min for plasmin only and 140.4 [IQR 111.3–156.6] min for pentamidine-treated groups, whereas this difference decreased at 800 nM plasmin to 11.2 [IQR 9.3–12.8] min for plasmin only and 31.6 [IQR 29.1–34.9] min for pentamidine. The maximum RFU observed in the pentamidine-treated group was lower across all plasmin concentrations. As shown in Figure 6C, at 200 nM and 350 nM, the difference between the maximum fluorescence is statistically significant (adjusted *p*-value < 0.05). Interestingly, the T90 remained relatively constant in the pentamidine-treated group, whereas the plasmin-only group showed a decrease in T90 with increasing plasmin concentration (Figure 6D). Group comparisons showed a significant difference in the time to reach 90% digestion, observed only at plasmin concentrations > 500 nM.

## 4. Discussion

Fibrinolysis is essential for maintaining hemostasis and vascular patency. Assays that can reproducibly quantify fibrinolysis phenotypes are central for managing bleeding disorders. Here, we have explored extending the previously developed fibrin annular clot assay to incorporate plasma from healthy donors. As shown in this study, plasma annular clots tested for fibrinolysis in the presence of plasmin exhibited a strong correlation with the maximum amplitude observed on TEG. The linear increase in maximum fibrinolysis rate with increasing plasmin concentration, observed in both fibrin and plasma annular clots, demonstrates the ability of the assay to accurately track fibrinolysis. The plasma annular clot assay described herein is a step towards developing a multi-assay platform for comprehensive fibrinolysis assessment. The adaptability of the assay enables modification and evaluation of the impact on clot dissolution kinetics in the presence and absence of various substrates, enzymes, and inhibitors to test pharmaceutical interventions on fibrinolysis with a patient’s own unique plasma clot composition. While all assays in this study used plasmin or pentamidine-treated plasmin added to the plasma annular clot system in buffer, the assay could also be performed with other pharmaceutical agents added to the annular clot system. This level of testing flexibility is advantageous, as it allows the assay to be modified to address different clinical questions under highly reproducible diverse assay conditions.

As for fibrinolysis kinetics observations of the pentamidine-treated group, we hypothesize that at lower plasmin concentrations, both the plasmin-only and pentamidine-treated groups converge on the T90 endpoint because the rate-limiting factor is the structural resistance of the highly cross-linked plasma clot, and the contribution of the inhibitor-mediated reservoir effect is insufficient to produce a measurable difference. Plasma contains a high concentration of serine protease inhibitors (SERPINs), which irreversibly inactivate serine proteases via a suicide-substrate mechanism [31]. At higher plasmin concentrations, in the absence of reversible inhibitors, we observe a decrease in the time to reach 90% digestion. Compared with the plasmin-only group, the pentamidine-treated group showed no change in the time to reach 90% digestion. Based on these kinetic observations, the current findings may be influenced by endogenous plasma SERPINs that are absent in the fibrin-only annular clot assay. In the pentamidine-treated group, if the inhibitor slows the initial cleavage rate, it extends the window during which plasmin is vulnerable to irreversible SERPIN-mediated inactivation, which can manifest as a net reduction in the total fibrinolytic rate but an extended digestion time at higher plasmin concentrations.

Viscoelastic assays reflect global, system-level fibrinolysis. Isolating the impact of any drug on fibrinolysis kinetics alone, independent of its interactions with coagulation proteins upstream, can be challenging to assess in a global fibrinolysis system. Additionally, inherited disorders of the fibrinolytic pathway have typically been difficult to diagnose using conventional methods due to the low sensitivity of these assays [32]. Patient plasma-based assays modified to increase system sensitivity can be an important step towards diagnosing bleeding conditions in the future. Recent advances in managing bleeding disorders have focused on using platelet-poor plasma and clot lysis to differentiate hyperfibrinolysis from shutdown subgroups, or on using detergent-incorporated clot lysis assays to distinguish fibrinolysis in trauma patients [33,34]. Plasma-based fibrin clot formation and lysis assays have also been developed to characterize septic shock in patients, and plasma clot-lysis parameters have shown a stronger association with coagulation and organ dysfunction, independent of fibrinogen and D-dimer levels [35]. Future studies using the plasma annular clot lysis assay will incorporate and compare key fibrinolytic inhibitors and patient-specific cohorts to validate the capacity of the assay to resolve pathway-specific impacts on fibrinolysis kinetics. Advances in fibrinolysis assays, either as a standalone assay or in addition to viscoelastic testing platforms, have the potential to improve health outcomes, specifically by screening for dysregulated fibrinolysis in patients with increased sensitivity. Fibrinolysis assays can significantly improve risk stratification across bleeding and thrombotic events, as in the case of trauma, obstetric hemorrhage, or cancer- associated coagulopathies.

## 5. Conclusions

In this study, we describe the incorporation of human-derived plasma into an established fibrin annular clot lysis assay platform. The adaptability of the annular clot lysis assay and its ability to dynamically track fibrinolysis provide an advantage for it to be used in further developing ex vivo fibrinolysis detection systems. With the ability of the system to track fibrinolysis reproducibly at different drug concentrations, this method can be further used in creating a multiplex platform for screening and advancing the preclinical development of thrombolytic drugs as well.

### Limitations

The authors acknowledge the limitations of the current study. The sample size of five males and five females limits the inferences about sex as a biological variable in this study. As fibrinogen concentration was not measured for each individual donor, multiple regression analysis was not performed to account for its potential confounding influence on the observed correlation between maximum fibrinolytic rate and TEG maximum amplitude, and both findings should be interpreted accordingly.

## Data Availability

The original contributions presented in this study are included in the article/Supplementary Material. Further inquiries can be directed to the corresponding author.

## Supplementary Materials

The following supporting information can be downloaded at: https://www.mdpi.com/article/10.3390/biom16070949/s1, Figure S1: All subject whole blood and plasma TEG traces; Figure S2: V_MFR_ for annular clot digestion observed at different volumes of plasmin solution addition at 500nM plasmin concentration; Figure S3: Normality assessment of the residuals before and after log transformation of residuals for (A) Fibrin, (B) Plasmin, and (C) Pentamidine-treated group, respectively; Figure S4: Baseline fibrinolysis rate observed for fibrin, plasma, and pentamidine-treated plasma clots. The maximum velocity observed across all samples was below 10 RFU/min, indicating minimal fibrinolysis in all tested clots when incubated without the addition of fibrinolysis enzyme; Table S1: TEG values for plasma samples; Table S2: TEG values for whole blood samples; Table S3: Annular clot values for all tested datasets.
