# Supplementary material for: Real-Time Fibrinolysis Monitoring of Plasma Annular Clots
Source: Biomolecules. 2026 Jun 26;16(7):949. doi: 10.3390/biom16070949 (PMC13406176; doi:10.3390/biom16070949)
Supplement: Supplementary file 1 [file biomolecules-16-00949-s001.zip › biomolecules-4364467-supplementary.pdf]

Supporting Information

Real-Time Fibrinolysis Monitoring of Plasma Annular Clots

Andres Prieto Trujillo [1,†], Anushri Umesh [1,2,†], Abigail Hall [1] and Nathan J. Alves [1,2,*]

1 Department of Emergency Medicine, Indiana University School of Medicine, 635 Barnhill Dr. Rm. 2063, Indianapolis, IN 46202, USA
2 Weldon School of Biomedical Engineering, Purdue University, West Lafayette, IN 47907, USA
* Correspondence: nalves@iu.edu
† These authors contributed equally to this work.

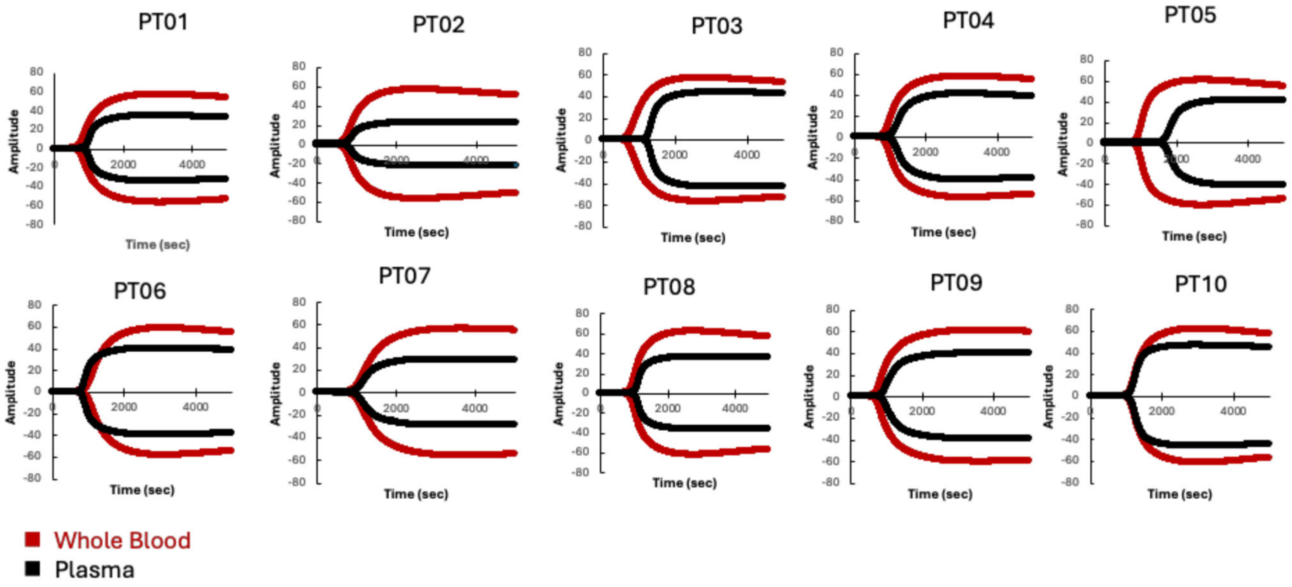

Figure S1. All subject whole blood and plasma TEG traces.

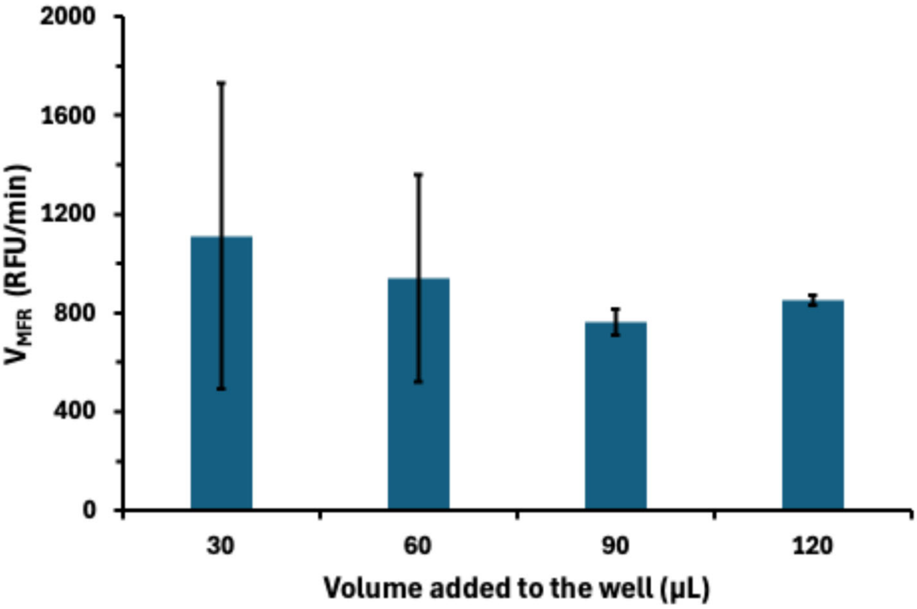

Figure S2. V_{MFR} for annular clot digestion observed at different volumes of plasmin solution addition at 500nM plasmin concentration.

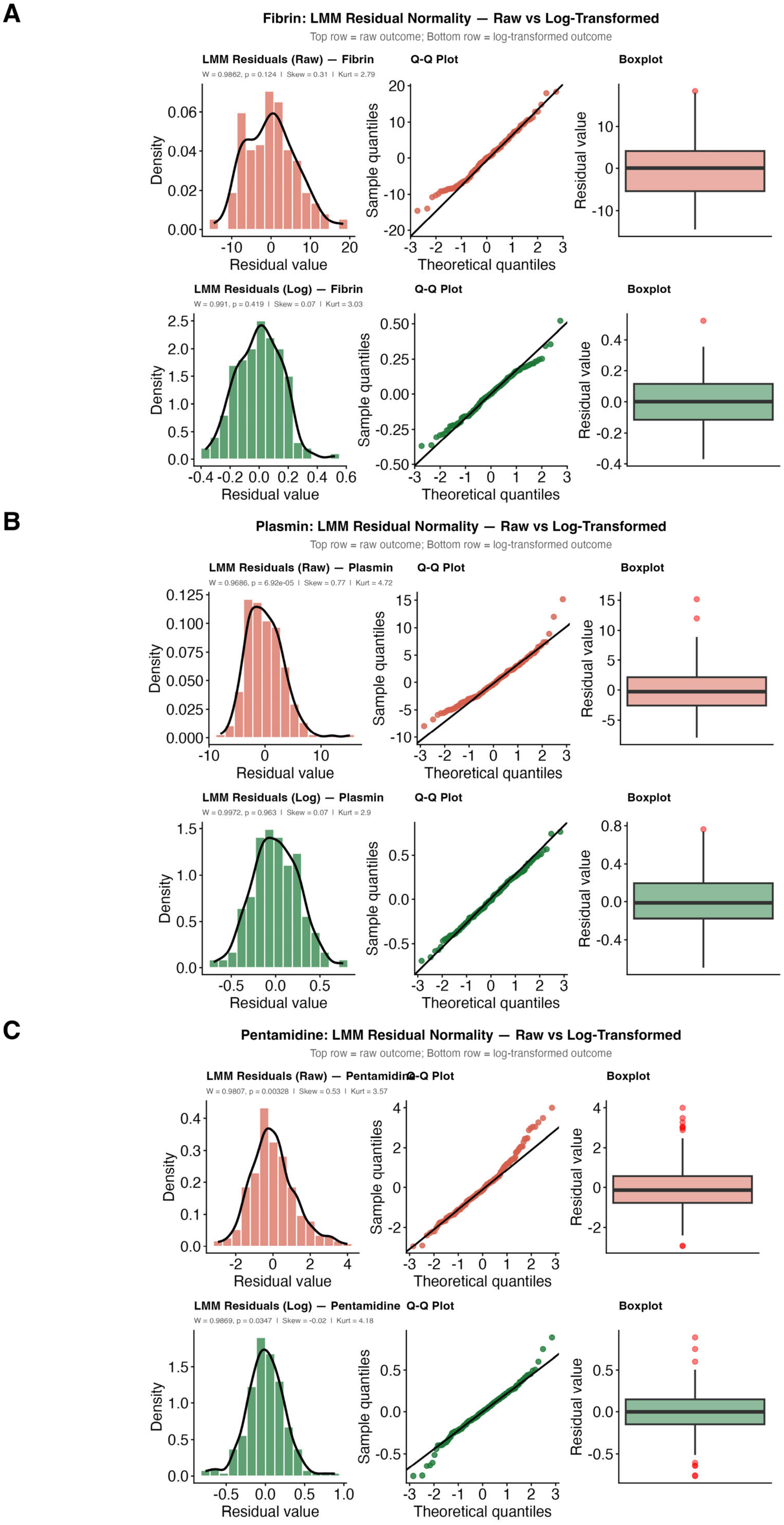

Figure S3. Normality assessment of the residuals before and after log transformation of residuals for (A) Fibrin, (B) Plasmin, and (C) Pentamidine-treated group, respectively.

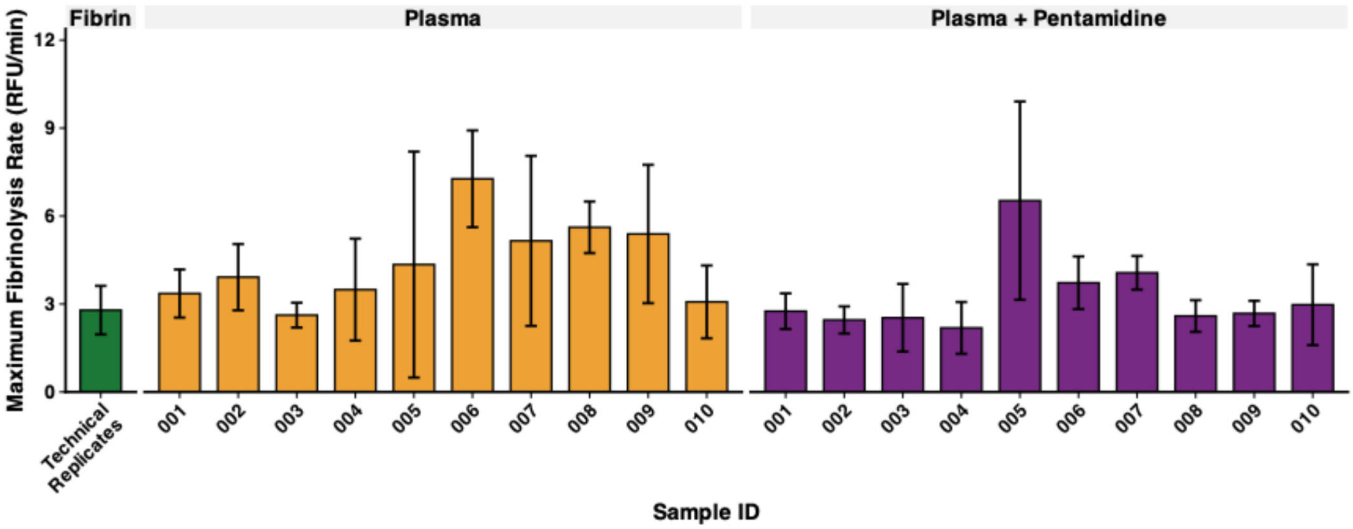

Figure S4. Baseline fibrinolysis rate observed for fibrin, plasma, and pentamidine-treated plasma clots. The maximum velocity observed across all samples was below 10 RFU/min, indicating minimal fibrinolysis in all tested clots when incubated without the addition of fibrinolysis enzyme.

Table S1. TEG values for plasma samples.

	Male (n=5)		Female (n=5)		Overall (n=10)	
	Mean ± SD	[Min, Max]	Mean ± SD	[Min, Max]	Mean ± SD	[Min, Max]
**Age**	26.6 ± 6.4	[20.0, 37.0]	25.0 ± 5.9	[19.0, 33.0]	25.8 ± 5.9	[19.0, 37.0]
**R time (min)**	14.78 ± 1.58	[13.10,17.20]	19.98 ± 4.37	[16.70, 27.40]	17.38 ± 4.14	[13.10, 27.40]
**K time (min)**	5.50 ± 3.26	[3.0, 11.20]	4.20 ± 1.77	[3.00, 7.20]	4.85 ± 2.57	[3.00, 11.20]
**Maximum amplitude (mm)**	34.08 ± 7.60	[21.20, 39.90]	38.34 ± 6.62	[28.50, 45.30]	36.21 ± 7.08	[21.20, 45.30]
**Alpha Angle**	38.02 ± 7.04	[31.60, 48.80]	40.08 ± 10.10	[24.40, 49.60]	39.05 ± 8.28	[24.40, 49.60]

Table S2. TEG values for whole blood samples.

	Male (n=5)		Female (n=5)		Overall (n=10)	
	Mean ± SD	[Min, Max]	Mean ± SD	[Min, Max]	Mean ± SD	[Min, Max]
**Age**	26.6 ± 6.4	[20.0, 37.0]	25.0 ± 5.9	[19.0, 33.0]	25.8 ± 5.9	[19.0, 37.0]
**R time (min)**	12.24 ± 1.84	[10.40,14.80]	14.44 ± 2.45	[10.90, 17.80]	13.34 ± 2.35	[10.40, 17.80]
**K time (min)**	4.14 ± 0.43	[3.70, 4.80]	3.86 ± 1.12	[2.80, 5.60]	4.00 ± 0.81	[2.80, 5.60]
**Maximum amplitude (mm)**	57.00 ± 1.48	[55.40, 59.10]	58.66 ± 3.07	[54.90, 61.50]	57.83 ± 2.44	[54.90, 61.50]
**Alpha Angle**	41.46 ± 3.29	[35.90, 44.20]	45.00 ± 6.68	[34.90, 51.70]	43.08 ± 5.36	[34.90, 51.70]

Table S3. Annular clot values for all tested datasets.

Dataset	Plasmin Concentration (nM)	Subjects	Max Digestion Rate (RFU/min)		FLU$_{200}$ (min)		T90 (min)		MaxFLU (Fluorescence units)	
			Median [IQR]	Mean +/- SD	Median [IQR]	Mean +/- SD	Median [IQR]	Mean +/- SD	Median [IQR]	Mean +/- SD
**Fibrin**	**0**		2.77 [2.35 - 3.1]	2.83 +/- 0.63	115.25 [58.88 - 136.88]	92.08 +/- 80.54	83.12 [62.25 - 94.56]	84.04 +/- 39.78	123.52 [97.26 - 154.44]	202.81 +/- 221.26
	**200**	6	21.51 [18.57 - 25.24]	20.83 +/- 5.52	14.12 [10.75 - 15.81]	13.9 +/- 4.34	134 [131.19 - 136.25]	134.2 +/- 3.48	2094.83 [1663.71 - 2232.51]	1944.73 +/- 418.76
	**350**	7	36.65 [25.61 - 38.8]	32.19 +/- 10.06	9.12 [7.88 - 11.33]	9.45 +/- 3.1	128.75 [125.19 - 133.56]	129.81 +/- 6.94	2515.01 [1960.3 - 2692.76]	2359.28 +/- 464.96
	**500**	7	46.71 [34.17 - 54.26]	43.38 +/- 12.89	7.25 [5.62 - 9.81]	7.71 +/- 2.79	117.75 [114.81 - 126.94]	121.07 +/- 9.9	3082.8 [2325.11 - 3236.72]	2814.37 +/- 533.23
	**650**	7	44.23 [37.7 - 50.67]	43.94 +/- 9.08	7.75 [5.75 - 8.88]	7.5 +/- 2.87	119.75 [112.5 - 122.19]	117.89 +/- 9.24	2970.27 [2426.94 - 3229.62]	2820.43 +/- 479.98
	**800**	7	60.49 [46.4 - 65.89]	55.89 +/- 12.23	7.5 [5.25 - 8.19]	6.96 +/- 2.36	114.38 [112.12 - 115.73]	115.17 +/- 5.42	3161.82 [2607.83 - 3267.06]	2937.15 +/- 413.67
**Plasma**	**0**		4.13 [3.39 - 5.33]	4.42 +/- 1.43	186.5 [166.62 - 210.08]	182.68 +/- 48.69	158.83 [121.94 - 182.19]	149.29 +/- 41.95	176.39 [160.37 - 204.17]	177.02 +/- 44.68
	**200**	10	5.18 [3.89 - 5.23]	4.88 +/- 1.44	52.31 [46.03 - 68.38]	59.99 +/- 25.38	205.81 [200.72 - 207.69]	203.06 +/- 9.74	829.85 [521 - 973.59]	751.82 +/- 281.87
	**350**	10	7.08 [6.89 - 7.58]	7.21 +/- 1.42	32.88 [25.46 - 34.19]	31.72 +/- 7.54	202.38 [195.5 - 204.91]	200.45 +/- 6.82	1016.21 [754.45 - 1196.39]	953.59 +/- 302
	**500**	10	11.02 [6.52 - 13.91]	10.7 +/- 4.12	16.88 [14.97 - 23.72]	19.25 +/- 5.42	167.5 [141.62 - 206.09]	165.65 +/- 48.42	906.44 [847.08 - 1228.19]	1020.86 +/- 242.56
	**650**	10	17.13 [10.85 - 20.05]	15.93 +/- 5.05	13.19 [11.22 - 16.22]	13.74 +/- 2.77	120.06 [97.12 - 171.53]	132.51 +/- 41.78	1161.43 [1019.12 - 1461.28]	1214.01 +/- 262.43
	**800**	10	23.82 [14.49 - 25.8]	21.05 +/- 6.49	11.19 [9.28 - 12.78]	11.48 +/- 2.66	90.4 [86.88 - 107.09]	102.03 +/- 26.72	1330.47 [1226.84 - 1584.39]	1392.3 +/- 300.73
**Pentamidine- treated**	**0**		2.75 [2.59 - 3.72]	3.33 +/- 1	133.5 [112.5 - 199.75]	163.67 +/- 91	228 [194.17 - 243]	219.31 +/- 34	149.53 [139.75 - 167.21]	153.11 +/- 30
	**200**	9	3.16 [3.07 - 3.6]	3.75 +/- 1.31	140.44 [111.28 - 156.56]	141.69 +/- 37.74	213.38 [201.25 - 228.62]	212.88 +/- 24.38	209.85 [175.09 - 234.52]	209.69 +/- 49.95
	**350**	9	3.9 [3 - 4.92]	4.48 +/- 2.01	58.62 [53.5 - 66.62]	77.61 +/- 48.92	228.5 [202.33 - 233.5]	221.61 +/- 24.79	414.07 [358.08 - 451.51]	407.84 +/- 109.11
	**500**	9	4.43 [3.97 - 5.17]	4.73 +/- 1.39	38 [35.5 - 45.12]	44.21 +/- 18.49	226.75 [214.5 - 228.25]	222.12 +/- 26.93	732.89 [722.89 - 847.12]	732.76 +/- 183.02
	**650**	9	5.49 [4.96 - 6.47]	5.69 +/- 1.14	31.88 [30.1 - 34.25]	35.16 +/- 11.74	241.7 [228.5 - 250.75]	237.09 +/- 19.9	1084.66 [948.31 - 1272.59]	1082.95 +/- 286.48
	**800**	9	6.02 [5.61 - 6.77]	6.32 +/- 1.08	31.62 [29.12 - 34.88]	33.16 +/- 10.15	242.17 [228.5 - 245.12]	238.02 +/- 16.98	1229.56 [1119.94 - 1372.18]	1215.03 +/- 255.9
